# Perioperative management including dual cell salvage in a Jehovah's Witness patient undergoing major urological surgery

**DOI:** 10.1002/ccr3.5098

**Published:** 2021-11-19

**Authors:** Thomas G. Smith, Irina Anastasescu, James M. Wight, Anicee Danaee, Rajesh Nair, Tim S. O'Brien

**Affiliations:** ^1^ Department of Anaesthesia Guy's and St Thomas' NHS Foundation Trust London UK; ^2^ Centre for Human and Applied Physiological Sciences King's College London London UK; ^3^ Department of Haematology Guy's and St Thomas' NHS Foundation Trust London UK; ^4^ Department of Urology Guy's and St Thomas' NHS Foundation Trust London UK

**Keywords:** caval tumor, cell salvage, Jehovah's Witness, major hemorrhage, renal tumor

## Abstract

Complex surgery associated with major hemorrhage presents particular risks for Jehovah's Witnesses who do not accept transfusion of blood products. Intraoperative use of two cell saver machines simultaneously can maximize the yield of salvaged blood from both the operative field and from washed surgical swabs and can potentially be life‐saving.

## Graphical abstract

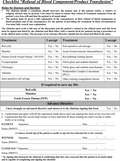

## 1 INTRODUCTION

Jehovah's Witnesses typically do not accept transfusion of red blood cells, fresh frozen plasma (FFP) and platelets based on their religious beliefs, although the use of fractionated blood derivatives and other interventions is at the discretion of the individual.^1^, ^2^ Importantly, many Jehovah's Witnesses will accept other products including cryoprecipitate, prothrombin complex concentrate, fibrinogen concentrate, recombinant blood products, and human‐derived topical hemostatic agents, and the majority will accept intraoperative cell salvage.^1^, ^2^

For patients facing major surgery associated with significant intraoperative bleeding, any contraindication to the use of blood products confers greater perioperative risk.^3^ However, surgical resection may be the only potentially curative treatment, as it is for renal tumors with extension into the inferior vena cava (IVC).^4^ Our institution is a quaternary referral center for surgical management of very large and otherwise complex renal and caval tumors, with current experience of more than 250 IVC cases, including 80 classified as level 3c (tumor thrombus extending above the diaphragm). Intraoperative bleeding can be high (up to 10 L in recent experience) requiring life‐saving transfusion of blood products. Here we present a case of an open radical nephrectomy and IVC exploration in a Jehovah's Witness patient where the perioperative outcome was favorable despite major bleeding. We particularly focus on unusual aspects of perioperative care that fall outside mainstream anesthetic experience but contributed significantly to this outcome.

## 2 REPORT

The patient was a 68‐year‐old man with a large renal tumor invading the IVC who had presented with clot retention. He was a Jehovah's Witness and was referred from a tertiary center where surgical management had been declined because the use of blood products was contraindicated due to patient refusal. His past medical history included diet‐controlled type 2 diabetes mellitus and well‐controlled epilepsy, with BMI 30 kg.m^−2^. Imaging showed a 13 cm right renal tumor with complex IVC involvement and heavy collateralization, with tumor thrombus extending into the retrohepatic IVC to the level of the main hepatic veins and into the contralateral renal vein, gross dilation of the IVC to a diameter of 4–5 cm, and non‐tumor thrombus obstructing the lower IVC. There was no distant metastatic disease. Resection required suprahepatic, infrapericardial vascular control and was anticipated to be challenging with potential for major hemorrhage, and the patient was already severely anemic^5^ with a hemoglobin concentration of 77 g.L^−1^.

**FIGURE 1:**
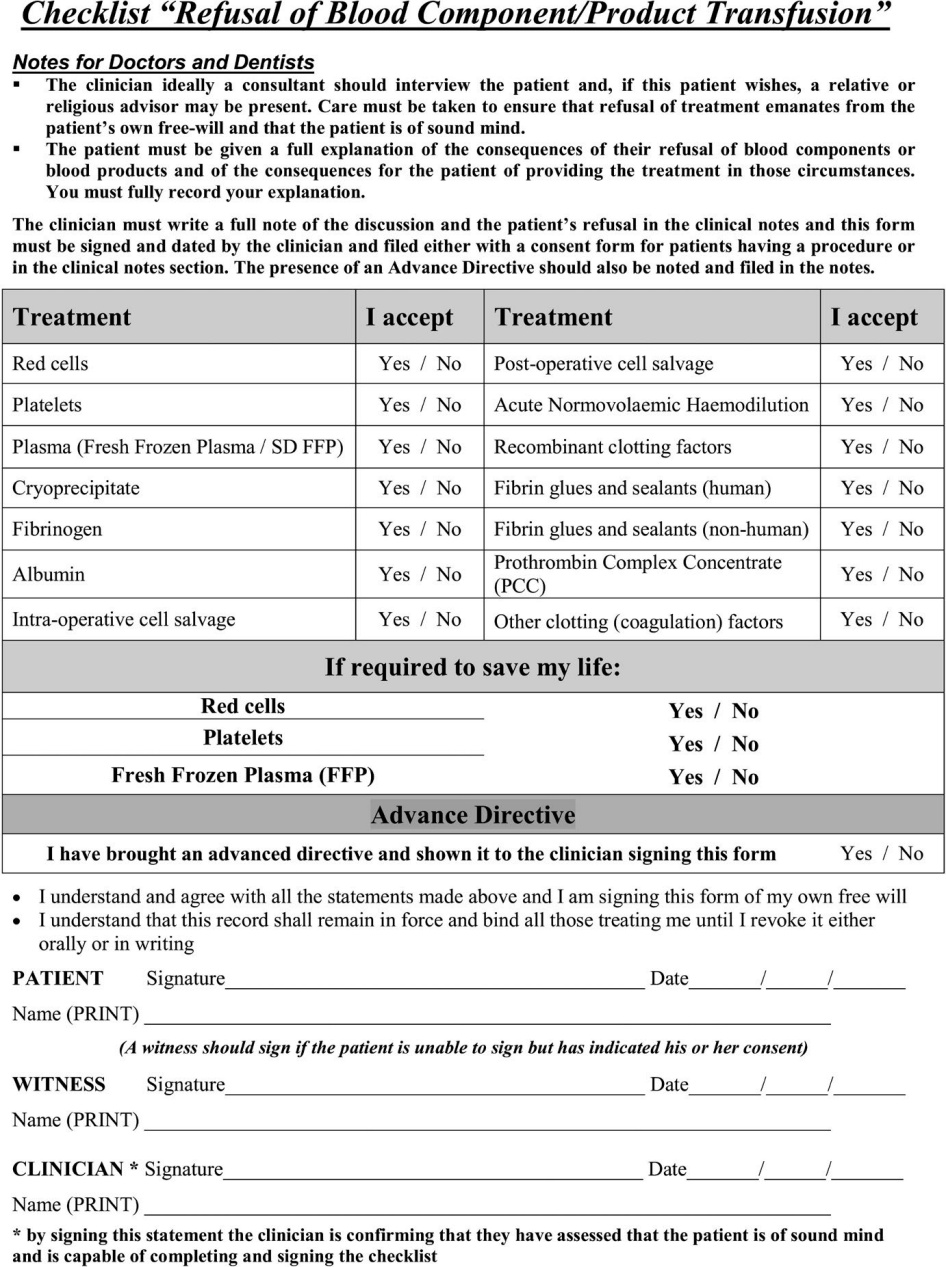
Refusal of blood component/product transfusion checklist. From the Guy's and St Thomas' NHS Foundation Trust clinical policy: “Clinical guidance for the management of patients refusing blood transfusion including Jehovah's Witness patients”

The patient confirmed that he refused to accept red blood cells, platelets or FFP. However, he accepted all other products and interventions including cell salvage and cryoprecipitate, as documented using our hospital's checklist (Figure 1). A meticulous plan for intraoperative management was developed by the surgical, anesthetic, and hematology teams while the patient's hemoglobin concentration was optimized over a period of 7 weeks with erythropoietin and maximum‐dose IV iron. This consisted of an initial dose of a long‐acting erythropoiesis‐stimulating agent (methoxy polyethylene glycol‐epoetin beta 125 mcg) followed by regular self‐administration of a short‐acting agent (epoetin beta 510,000 units in total), in combination with ferric carboxymaltose (4 g in total). The hemoglobin concentration reached 110 g.L^−1^ by the time of surgery. As part of the patient's hematological management, an explicit and personalized “Individual Treatment Plan” for emergency intraoperative hemostasis was formulated comprising administration of cryoprecipitate, then prothrombin complex concentrate (30 units.kg^−1^ on hand), then fibrinogen concentrate (3 g) as a last resort.

The operating theatre team included three consultant surgeons and two consultant anesthetists. Two cell saver machines were prepared to allow for dedicated cell salvage from washed surgical swabs. The patient had a spinal anesthetic with intrathecal diamorphine followed by general anesthesia using volatile maintenance. Arterial and central venous catheters and a Vascath were inserted and acute hypervolemic hemodilution was achieved by administration of 1 L warmed crystalloid at induction.^2^, ^6^ Tranexamic acid 1 g IV was administered at induction and six‐hourly afterwards. A low‐dose noradrenaline infusion (<1 mcg.kg^−1^.min^−1^) was titrated throughout the case. Surgery was performed via a Mercedes Benz incision and the duration of the case was approximately 10 h. The intraoperative course was difficult with periods of rapid hemorrhage, especially from the junction of hepatic vein and IVC and from high pressure collaterals between the kidney and liver. Estimated blood loss was approximately 4 L and the lowest hemoglobin concentration measured intraoperatively was 35 g.L^−1^. The patient received more than 1 L of concentrated red blood cells returned from cell salvage, as well as 8 units of cryoprecipitate, 7 L of crystalloid, 1 L of Geloplasma and prothrombin complex concentrate (15 units.kg^−1^).

At the end of the case, the patient was stable with hemoglobin concentration 53 g.L^−1^ and minimal noradrenaline requirements and was transferred to the critical care unit following extubation in the operating theatre. He was discharged to the ward on day 2 and home on day 6 postoperatively with hemoglobin concentration 78 g.L^−1^, the same as when he was referred. Histology subsequently showed a grade 4 clear cell renal cell carcinoma, and hemoglobin concentration normalized within 3 months.

## 3 DISCUSSION

Bleeding associated with open radical nephrectomy and IVC exploration carries a risk of morbidity and mortality even without limitations on the use of blood products. When transfusion is contraindicated, the risks must be mitigated as much as possible by careful optimization and preparation. Many factors contribute to a favorable outcome in complex cases such as this – here we concentrate on key points relating to anesthetic management and perioperative care.

This case illustrates the use of the Association of Anaesthetists' guidelines for anesthesia and perioperative care for Jehovah's Witnesses,^1^ and additionally reports some more detailed aspects of management that do not feature in these guidelines. Our approach was also informed by local experience and hospital policies as well as other published literature, and was developed in discussion between the anesthetic, surgical, and hematology teams. Aggressive preoperative optimization of hemoglobin achieved a large increment in hemoglobin concentration of approximately 40%, without which surgery may not have been survivable. The use of erythropoietin and iron therapy in this context is well established^7^ and introduces a tension between the need to delay surgery for optimization and the need to expedite definitive treatment. Balancing these competing imperatives is a matter of clinical judgment that depends on a patient's particular circumstances. This also requires a sense of urgency, with anemia therapy ideally initiated as soon as possible after diagnosis. From the current case, the importance of commencing treatment immediately, and the potential for influencing acute outcomes through such a large increment, should be emphasized.

A less well known but indispensable aspect of hematological management was the preparation of a perioperative “Individual Treatment Plan” by a hematologist subspecializing in hemostasis and thrombosis. This is common practice for patients with bleeding disorders such as hemophilia who require surgery but is not commonly used in other situations. Borrowing this “hemophilia‐style” approach, our plan consisted of a patient‐specific protocol encompassing each phase of care including preoperative optimization, postoperative anticoagulation and, crucially, a “worst‐case scenario” intraoperative plan. Key personnel including pharmacy, laboratory and transfusion staff were involved in preparing this advance plan for emergency intraoperative hemostasis, which enabled immediate administration of cryoprecipitate (as the mainstay) and prothrombin complex concentrate at vital moments during surgery (fibrinogen concentrate was held in reserve and not required). Close liaison between the lead anesthetist and hematologist continued during the surgery itself and, with the benefit of specialist advice, cryoprecipitate was notably used quite liberally, almost as a surrogate for FFP. This was highly effective, with no evidence of coagulopathy or hypercoagulability throughout, and complemented the multiple surgical techniques used to achieve hemostasis (suture ligation plus Ligaclips and extensive use of Floseal, Surgicel Fibrillar and argon beam coagulation).

Cell salvage was a very important factor in this case, in which intraoperative blood loss equated to approximately two‐thirds of the patient's calculated circulating blood volume of circa 5.6 L.^8^ Anesthetic guidelines advise that cell salvage should be considered for all surgical procedures in Jehovah's Witness patients if blood loss >500 mL is possible.^1^ This case has highlighted the potential benefits of simultaneously using two cell saver machines, which has been reported for open thoracoabdominal aortic aneurysm repair^9^, ^10^ but, to our knowledge, does not feature in current guidelines or literature relating to major surgery in Jehovah's Witness patients.

In this case, bloody surgical swabs were soaked in normal saline and gently washed by a dedicated scrub nurse. It was essential to reclaim this blood while ensuring that the availability of maximal suction power at the operative field was not compromised, precluding the connection of multiple suction lines to a single cell salvage machine. Instead, a separate cell saver was used for the wash fluid from swabs, preserving maximal suction for the surgical field from the primary machine. The use of dual cell salvage is unusual and falls outside mainstream anesthetic experience, but was instrumental in maximizing the yield from salvaged blood and consequently, alongside other factors, was critical to the favorable perioperative outcome and indeed the patient's survival. As a relatively straightforward technique that can make a potentially life‐saving difference, we suggest it may be prudent to consider the option of dual cell salvage in Jehovah's Witness patients undergoing similarly high‐risk surgery.

In summary, it is rare to undertake complex surgery associated with major hemorrhage in a patient where the use of blood products is contraindicated, and this case illustrates the benefits of careful, patient‐specific, multidisciplinary planning in perioperative management. It also highlights specific elements that we believe made an important difference to the outcome including early and aggressive optimization of hemoglobin concentration, the use of a hemophilia‐style treatment plan to guide emergency intraoperative hemostasis, expanded use of cryoprecipitate in place of FFP, and the use of more than one cell saver machine. Dual cell salvage could be considered more widely as part of intraoperative blood management strategies and may warrant consideration for inclusion in future guidelines.

## CONFLICTS OF INTEREST

No external funding or competing interests declared.

## AUTHOR CONTRIBUTIONS

TGS, IA and JMW were responsible for anesthetic management. AD was responsible for hematological management. TSOB and RN were responsible for surgical management. TGS drafted the manuscript. All authors were responsible for critical revision of the manuscript for important intellectual content.

## ETHICAL APPROVAL

Written informed consent of the patient was obtained for publication.

## CONSENT

Published with the written consent of the patient.

## Data Availability

Data sharing is not applicable.
